# Cold air plasma improving rheumatoid arthritis via mitochondrial apoptosis pathway

**DOI:** 10.1002/btm2.10366

**Published:** 2022-07-01

**Authors:** Chengbiao Ding, Leying Ni, Qi Liu, Chenxu Zhou, Guomin Wang, Paul K. Chu, Zhengwei Wu

**Affiliations:** ^1^ School of Nuclear Science and Technology University of Science and Technology of China Hefei China; ^2^ Department of Rehabilitation Medicine The Second Hospital of Anhui Medical University Hefei Anhui China; ^3^ Department of Physics City University of Hong Kong Kowloon, Hong Kong China; ^4^ Department of Biomedical Engineering City University of Hong Kong Kowloon, Hong Kong China; ^5^ Department of Materials Science and Engineering City University of Hong Kong Kowloon, Hong Kong China; ^6^ CAS Key Laboratory of Geospace Environment University of Science and Technology of China Hefei China; ^7^ Institute of Advanced Technology University of Science and Technology of China Hefei China

**Keywords:** cold air plasma, mitochondrial apoptosis pathway, rheumatoid arthritis, ROS

## Abstract

Rheumatoid arthritis (RA) has plagued physicians and patients for years due to the lack of targeted treatment. In this study, inspired by the commonality between rheumatoid arthritis fibroblast‐like synoviocytes (RA‐FLS) and cancer cells, the therapeutic effects of cold air plasma (CAP) on RA are studied systematically and thoroughly. In/ex vivo results show that CAP with the proper dosage significantly relieves symptoms including synovial hyperplasia, inflammatory infiltration, and angiogenesis and eliminates the root cause by triggering the self‐antioxidant capability of the surrounding tissue. The mechanism on the molecular and cellular level is also revealed that the spontaneous reactive oxygen species (ROS) cascade induces the mitochondrial apoptosis pathway on RA‐FLS. This study reveals a new strategy for targeted treatment of RA and the mechanistic study provides the theoretical foundation for future development of plasma medicine.

## 1 INTRODUCTION

Rheumatoid arthritis (RA) is an autoimmune disease characterized by intraarticular synovial hyperplasia and inflammatory cell infiltration. Synovial proliferation can lead to bone and cartilage destruction of the joint and such damage can eventually result in bone erosion and joint deformity, from which patients suffer much.^1^, ^2^, ^3^ Current treatment of RA depends mainly on oral systemic medications aiming to improve the disease conditions and control symptoms. The adverse effects remain a hard nut to crack, (1) first‐line drugs such as nonsteroidal anti‐inflammatory drugs (NSAIDs) are associated with frequent side effects such as nausea, abdominal pain, ulcer, and gastrointestinal bleeding, while corticosteroids are associated with adverse events including osteoporosis, weight gain, diabetes, and immunosuppression^4^, ^5^; (2) second‐line drugs such as methotrexate can be the culprit of drug‐related cirrhosis and bone marrow deterioration^6^; (3) biological agents, albeit possessing better targeting ability in the treatment of RA, have unwanted side effects including increased risk of infection and common neurological effects such as multiple sclerosis and lymphoma.^7^, ^8^ Hence, RA has plagued physicians worldwide due to its lack of efficient and targeted treatment.

As early as 1550 BC, cautery was a mysterious and ancient method considered complementary to the treatment of complex diseases. In the 18th and 19th centuries, cautery was reported in Chinese and Indian medicine to improve joint pain in RA.^9^ Because of the inability to apply cautery to the intra articular and the inability to control the dose, cautery did not make substantial progress in the treatment of RA. In modern medicine, low‐temperature plasma radiofrequency ablation (LTPRA) has similar characteristics to cautery. The principle of operation is to ablate the local tissue around the electrode by evaporating it with radiofrequency energy, which seems to be a potential method to act on the joint synovium.^10^ However, LTPRA inevitably damages the healthy tissue while removing the diseased tissue, and it is difficult for LTPRA to realize the cooperation of visualization system and water supply system in a narrow joint space, which is still not a method to solve RA joint lesions.^11^

Recent studies have provided important clues concerning joint‐targeted therapy of RA. RA‐FLS are the core effector cells of knee joint synovium. The activated RA‐FLS cells are the lesion center of RA synovial proliferation.^12^ RA‐FLS is extremely similar to tumor cells.^13^ On the one hand, the defect of RA‐FLS apoptosis originates from aberrant proliferation and transformation in tumor samples, which shows similar invasiveness and adhesivity. On the other hand, the imbalance between antiapoptotic and proapoptotic molecules is an important cause for RA‐FLS antiapoptosis.^14^, ^15^ The above commonality between RA‐FLS and tumor cells inspires the attempt to treat RA referring to the potential anticancer strategy.

Cold air plasma (CAP) has been widely explored in the clinical as physical treatment for tumors. Although the reactions between plasma and cells are still not well understood, ROS representing oxygen‐containing free radicals in cells including superoxide radical (O_2_•−), hydrogen peroxide (H_2_O_2_), hydroxyl radical (OH•), peroxynitrite (ONOO−), nitric oxide (NO), and so on in the plasma are considered to be important factors affecting the functions of living cells.^16^ CAP induces cytotoxic reaction in many cell lines by regulating the intracellular ROS levels.^17^ In this respect, CAP effectively modifies glycated GPx, increases the enzymatic activity, and reduces oxidative stress in diabetic mice.^18^ In addition, CAP is used to induce apoptosis in glioblastoma, cervical cancer, and breast cancer cells.^19^ Therefore, CAP is expected to inhibit abnormal cell activation such as RA‐FLS. However, the preclinic feasibility of using a plasma to treat RA and whether it can regulate oxidative stress or promote apoptosis of RA‐FLS via the generation and delivery of chemically reactive free radicals have not been reported.

In this study, using an animal model, CAP is introduced into the knee joint cavity of rats. The therapeutic effects of CAP on diseased synovium are studied by HE staining of pathological sections and monitoring the ultrasonic blood flow changes in the joint cavity and level of antioxidant enzymes in the synovium before and after the treatment. On the cellular and molecular level, the effects of CAP on the characteristics of tumor‐like cells, such as apoptosis, proliferation, invasion, and migration of RA‐FLS, are evaluated. The effects of CAP on pathological synovial effector cells RA‐FLS are further assessed by apoptotic protein monitoring, intracellular ROS content measurement, mitochondrial membrane potential determination, and electron microscopy.

## 2 RESULTS

### 2.1 CAP devices and plasma characterization

**FIGURE 1:**
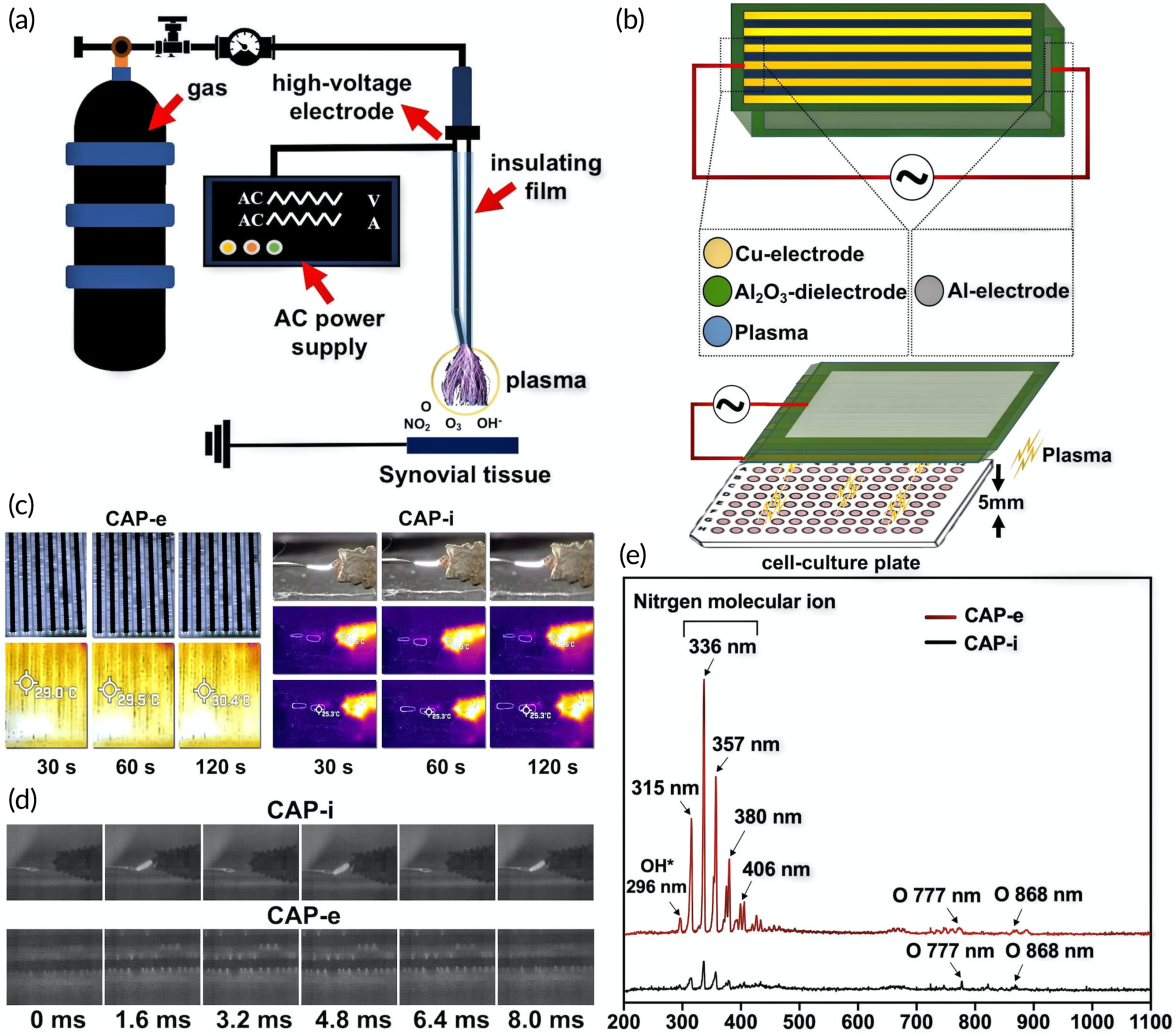
Illustration of cold air plasma (CAP) devices and physical characteristics of plasma. (a) Schematic diagram of CAP‐i device. (b) Schematic diagram of CAP‐e device. (c) The infrared thermometer measures the working temperature of CAP‐i and CAP‐e device at different times. (d) The discharge image was recorded by means of a high‐speed camera device (phantom) with 600 frames per second. (e) Emission spectra of CAP‐i and CAP‐e between 200 and 1100 nm

The CAP devices required for the in vivo and ex vivo experiments were designed and fabricated according to the target application. CAP used to intervene with the joint cavity of rats (CAP‐i) is shown in Figure 1a. The voltage difference between the puncture needle and tissue ionizes the air to generate the plasma, which is brought to the vicinity of the intraarticular synovium. The advantage of this design is to avoid the direct interaction between the electrode and intraarticular synovium. In addition, the cathode is connected to the rats at a zero potential and the device is operated at a peak current of only 4 mA, which is so small that there is very little tissue damage. The ex vivo experiments are conducted in RA‐FLS using the plasma device (CAP‐e) shown in Figure 1b, which is designed individually to achieve high‐throughput treatment of 96‐well cell culture plates. The detailed fabrication process of the device can be found elsewhere.^20^ Although the devices for the in vivo and ex vivo studies vary in appearance, they share key parameters such as the operating temperature, discharge form, and excited‐state generation. The temperature of the plasma generated by these two devices is monitored by an infrared thermometer as shown in Figure 1c. After the CAP‐e acts for 30 s, the temperature of the discharge panel is 29.0°C, and increases gently to 30.4°C after 120 s. When the CAP‐i is discharged to 120 s, the temperature of the arc column is maintained at 25.3°C and the temperature of the dielectric layer does not exceed 32.6°C. These results indicate that both devices establish a working environment with a temperature less than 40°C thus ensuring the safety of cells and tissues. The moderate working temperature can be explained by the pulse discharge form of the CAP device. The discharge of both CAP‐i and CAP‐e device is discontinuous and periodically pulsed thereby limiting the temperature rise. The chemical composition of the plasma is determined by optical emission spectroscopy (OES).^21^

This technology captures the unique emission spectrum of discharge plasma by using the characteristic emission spectra of different atoms or molecules. As shown in Figure 1e, the high intensity peaks at 315, 336, and 380 nm in the UV–vis spectrum are related to the second positive system of N_2_ and the first negative system of $N_{2}^{+}$.^22^ The characteristic emission peaks of atomic oxygen appear at 777 and 845 nm.^23^ These two substances correspond to ionic nitrogen and oxygen respectively, which represent the main part of the air. Moreover, the peak of OH* is observed at 296 nm for both devices.^24^ The above results fully illustrate the physical and chemical consistency of the discharge generated by both CAP‐i and CAP‐e.

### 2.2 Establishment of the animal model

**FIGURE 2:**
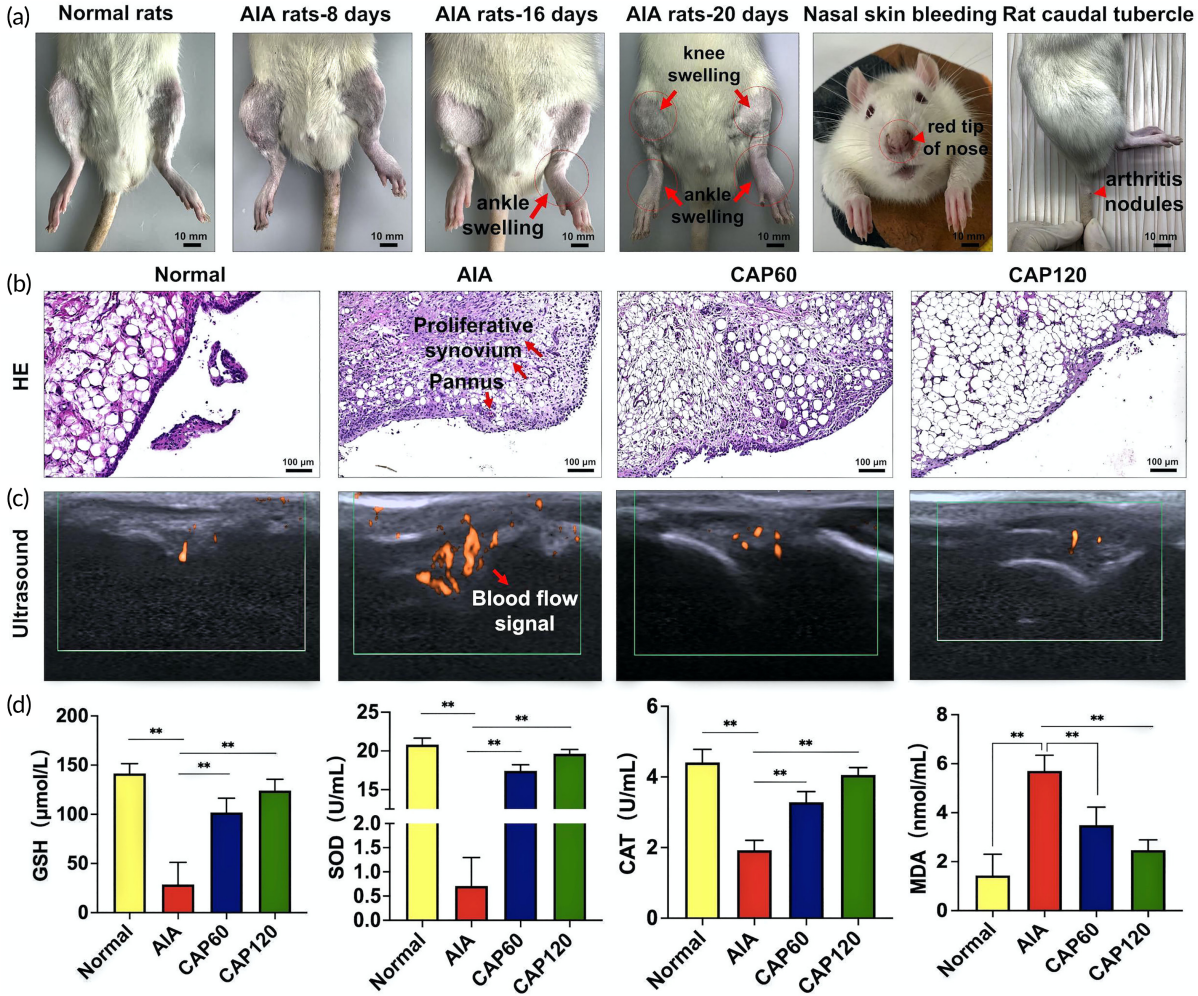
Cold air plasma (CAP) in vivo treatment can reduce the inflammation and proliferation of synovium in adjuvant‐induced arthritis (AIA) rats, inhibit blood flow and improve oxidative stress indicators. (a) Establishment of AIA rat model. (b) Changes in HE staining of the synovial tissue of the knee joint in each group of rats. (c) Changes in ultrasound blood flow in the synovial membrane of the knee joint in each group of rats. (d) Changes of oxidative stress indexes before and after treatment, ** indicates statistical differences: **p < 0.01

The adjuvant‐induced arthritis (AIA) model is established by injecting complete Freund's adjuvant into the left toes of the Sprague Dawley (SD) rats. Compared to other models, the adjuvant‐induced model has a higher success rate of arthritis and more pronounced joint symptoms thus better satisfying the need of CAP for targeted arthritis therapy.^25^ As shown in Figure 2a, the ankles of the rats in the normal group are relatively thin, while the ankles and knee joints of the rats in the AIA group swell gradually with time. In details, the contralateral knee joints swell after modeling for 16 days and then obviously 20 days later. In addition, the noses of rats are red, swollen, and broken, and arthritis nodules appear from the root of the tail. The morphology proves that the rat AIA model is established successfully.

### 2.3 CAP is effective in eliminating the symptoms of RA

Synovial hyperplasia, inflammatory infiltration, and angiogenesis as the specific pathological features for RA are determined by hematoxylin–eosin (HE) staining and ultrasonic imaging. As shown in Figure 2b, two to three layers of synovial intimas can be observed from the normal group with the epithelial cells arranged neatly without inflammatory cell infiltration. In contrast, in comparison with the normal group, the synovial tissues of the AIA group proliferate significantly and many inflammatory cells are infiltrated. After treatment by CAP for 60 s (CAP60), inflammatory cell infiltration eases, but synovial hyperplasia is hardly relieved. Nevertheless, an encouraging result is observed after plasma treatment for 120 s (CAP120) that both inflammatory infiltration and synovial hyperplasia are eliminated consequently rescuing the synovial tissue to the state similar to the normal group. Vascular proliferation as another pathological feature of RA is also attenuated by CAP treatment (Figure 2c). When monitoring the blood flow near the synovial tissue, a small color Doppler signal is seen from the normal group, while three big color Doppler signals taking up larger areas appear from the AIA group, indicating serious angiogenesis. The number and area of the color Doppler spots decrease obviously after the plasma treatment. The CAP120 group shows only two small color Doppler spots similar to the normal group representing highly improved vascular conditions. To summarize, CAP120 shows preferable therapeutic effects on RA by alleviating the symptoms of synovial hyperplasia, inflammatory infiltration, and angiogenesis.

### 2.4 CAP improves the root cause of RA by triggering the self‐antioxidant capability of tissues

Oxidative stress to a certain extent is the root cause for morbidity of RA and the antioxidant ability of the tissue determines the recovery from RA. Glutathione (GSH), superoxide dismutase (SOD), and catalase (CAT) as the critical catalyst pertaining to the scavenging of ROS and malondialdehyde (MDA) as the product of peroxidation marking the damage are quantitatively analyzed (Equations 1 and 2, respectively).^26^

(1) $$O_{2}\cdot \overset{\mathrm{SOD}}{\to }H_{2}O_{2}\overset{\mathrm{CAT}/\mathrm{GSH}\;}{\to }H_{2}O+O_{2}$$

(2) $$O_{2}∙/H_{2}O_{2}\;\overset{\;\text{Lipid peroxidation}\;}{\to }\;\mathrm{MDA}$$

As shown in Figure 2d, the concentration of SOD in the AIA group is at a low level (0.7 U/ml). After the plasma treatment, SOD in the tissue increases to 20 U/ml similar to the normal group indicating efficient clearance of $O_{2}∙$. A similar trend is also observed from the other two catalysts compared to the AIA group. CAP120 increases the concentrations of CAT and GSH by two and five times, respectively, to the similar level as the normal group inhibiting accumulation of H_2_O_2_. The results show that plenty of catalysts exist in the CAP‐treated tissue thus facilitating consumption of ROS, reducing oxidative damage to the tissue, suppressing the development of RA. This is confirmed by the reduced concentration in MDA from 5.7 nmol/ml for the AIA group to 1.4 nmol/ml for the CAP120 group as proof that little peroxidation damage takes place and RA is restrained after the CAP treatment. In short, CAP changes the environment in the joint by triggering the self‐antioxidant capability and eliminating the root cause for RA.

### 2.5 CAP inhibits the viability and proliferation of single RA‐FLS

**FIGURE 3:**
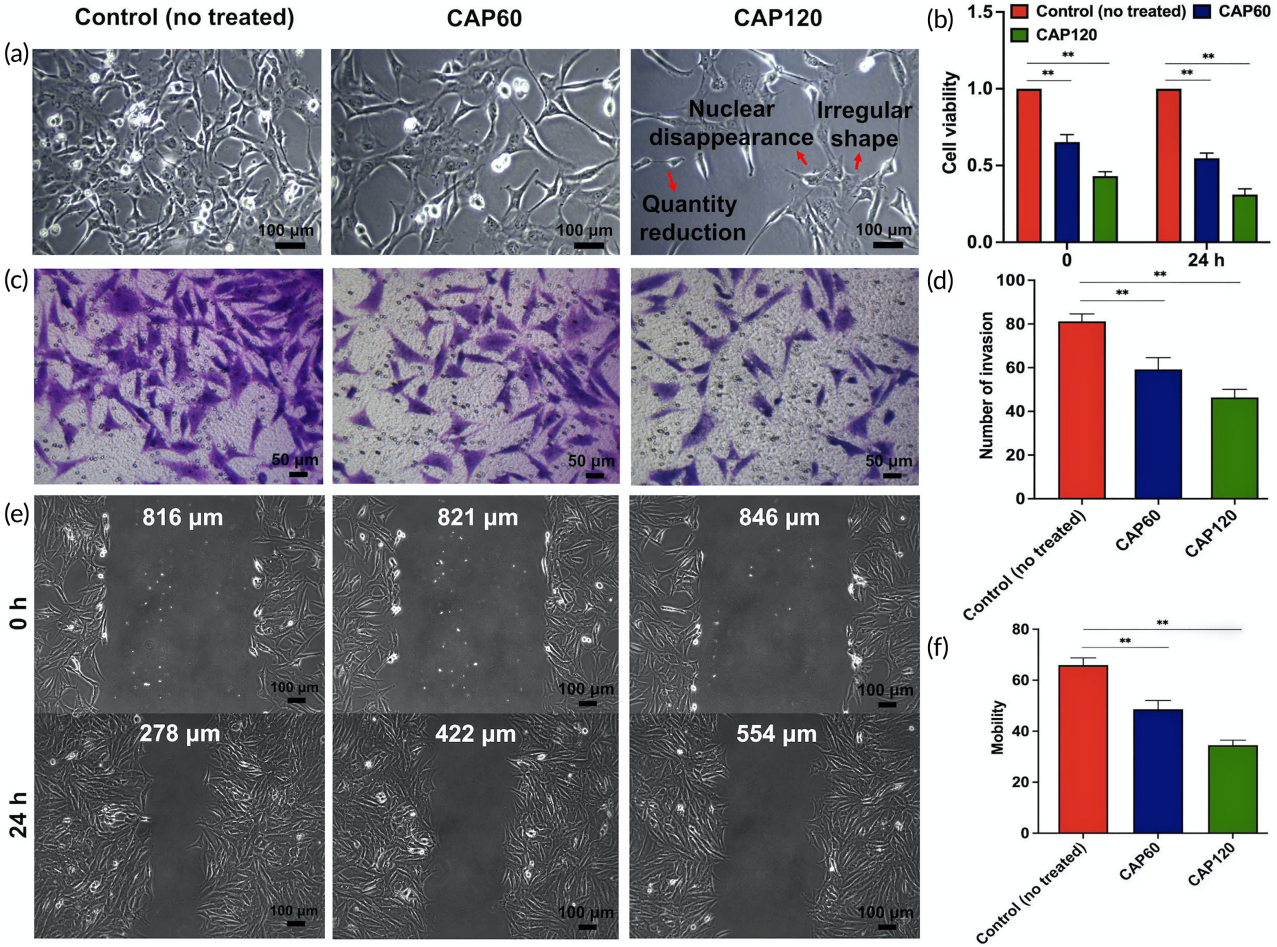
Cold air plasma (CAP) treatment ex vivo can reduce the cell viability, invasion, and migration of rheumatoid arthritis fibroblast‐like synoviocytes (RA‐FLS). (a) Cell morphology was observed by inverted microscope (magnification: 100×) at different time points of CAP treatment. (b) CCK‐8 assay of cell viability in each group. ** indicates statistical differences: **p < 0.01. (c) Experimental pictures of RA‐FLS invasion in each group. (d) Experimental pictures of RA‐FLS invasion in each group. (e) Pictures of RA‐FLS migration distance at 0 and 24 h after CAP intervention in each group. (f) Statistics of cell migration rate in each group. ** indicates statistical differences: **p < 0.01.

The morphology of RA‐FLS before and after CAP intervention was observed under the microscope. As shown in Figure 3a, the cells in the control (no treated) group have a fusiform shape with large and round nuclei located in the center. The cell number in CAP60 decreases, while the morphology changes mildly. With prolonged CAP treatment, the cells have a long and fusiform shape with more intercellular space and some of the nuclei even disappear. The viability and proliferation of cells are quantified by the CCK‐8 test (Figure 3b). The survival rates of the CAP60 group and CAP120 group are 65.3% and 43.1%, respectively, which are significantly lower than those of the control group. The difference becomes larger after culturing for 24 h. These results indicate that both the viability and proliferation of RA‐FLS are inhibited by CAP and so development of RA is prevented from the macroscopic perspective.

### 2.6 CAP obstructs the invasion and migration of RA‐FLS to the surrounding tissues and environment

RA‐FLS shows “tumor‐like” properties with the invasive and migratory behavior, which accelerates exponentially disease deterioration.^27^ The migratory behavior is evaluated by the scratch assay. The scratch healing speed of the control (no treated) group is faster as shown by the distance between the edges shortening significantly after 24 h. The healing speed decreases gradually after the CAP treatment as reflected by the wider distance between the edges in CAP60 and CAP120, which is observed from the figure and quantitatively indicated by halved migration rates in CAP120 compared with the control (no treated) group (Figure 3e,f). Subsequently, the transwell analysis is carried out to study whether CAP affects invasion of RA‐FLS (Figure 3c). Invasion in the CAP groups is weaker than in the control (no treated) group and CAP120 decreases the invasion density by a larger degree compared to CAP60 (Figure 3d). Adenosine triphosphate (ATP) produced by mitochondria provides energy for cell proliferation, migration, and invasion.^28^ Mitochondrial membrane potential (MMP) is a prerequisite to maintaining the mitochondrial oxidative phosphorylation and ATP production. Studies have shown that low levels of MMP can inhibit cell proliferation and migration,^29^ and the decrease of MMP is widely considered to be one of the earliest events in the process of apoptosis.^30^ Therefore, it is proposed that inhibition of tumor‐like features of RA‐FLS is caused by CAP induced mitochondrial damage and apoptosis, which are confirmed by the ex vivo results.

### 2.7 CAP triggers apoptosis of RA‐FLS

**FIGURE 4:**
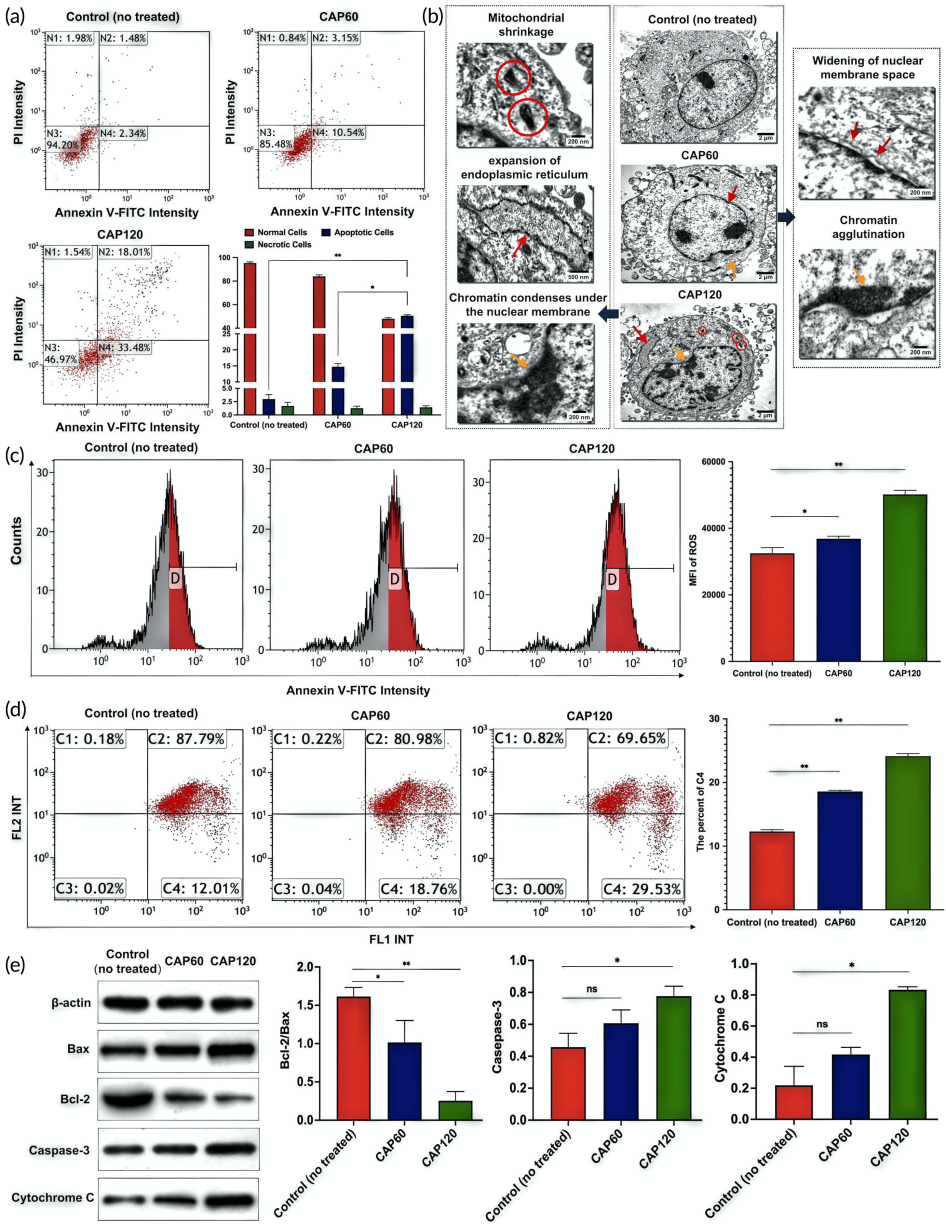
Changes of apoptosis, morphology, intracellular reactive oxygen species (ROS) content, mitochondrial membrane potential (MMP) and related apoptotic proteins in rheumatoid arthritis fibroblast‐like synoviocytes (RA‐FLS) before and after cold air plasma (CAP) intervention ex vivo: (a) Effect of CAP on apoptosis of RA‐FLS cells. Include the ratios of necrotic, apoptotic, and viable cells of each group within 24 h after CAP intervention and the apoptosis indexes of RA‐FLS cells of each group. * and ** indicates a significant difference between the groups, equal to *p < 0.05 and **p < 0.01. (b) Cell morphology at different time of the CAP treatment observed by transmission electron microscopy. (c) ROS content and statistical difference in the control group (untreated), the CAP60 group, and the CAP120 group, ** indicates a significant difference between the groups (*p* < 0.01). (d) JC‐1 fluorescence intensity in each group, C2 represents the proportion of cells in good condition, and C4 represents the proportion of cells with reduced membrane potential, * and ** indicates a significant difference between the groups, equal to *p < 0.05 and **p < 0.01. (e) Western blot detected the expressions of Bcl‐2, Bax, caspase‐3 and cytochrome c in RA‐FLS cells; Bcl‐2/Bax ratio; Relative expression of caspase‐3 protein; relative expression of cytochrome c protein. * and ** indicate that there are significant differences between the two groups, which are equal to *p < 0.05 and **p < 0.01 respectively. “ns” indicates that there is no difference between the two groups, which is equal to ^ns^ *p* > 0.05. Before and after CAP intervention, there was no significant difference in the protein content of activated caspase‐3 between CAP60 group and control (no treated) group (*p* = 0.095), and there was no significant difference in the protein content of cytochrome c between CAP60 group and control (no treated) group (*p* = 0.131).

The morphology of the cells obtained by transmission electron microscopy (TEM) imparts qualitative information of cell progression. As shown in Figure 4b, the nuclear membranes in the control (no treated) group are intact. After treatment by CAP for 60 s (CAP60), the nuclear membrane gap widens (red arrows in Figure 4b) and chromatin agglutinates (orange arrows in Figure 4b) indicating early apoptosis. Typical apoptotic phenotypes including nuclear chromatin pyknosis, edge aggregation, mitochondrial shrinkage, and endoplasmic reticulum expansion are observed after the treatment for 120 s (CAP120). The apoptosis status of treated RA‐FLS is analyzed quantitatively by flow cytometer (Figure 4a). In the control group, 94.2% of the cells are viable with only 3.82% marked as apoptotic. The CAP treatment increases the ratio of the apoptosis cells to 13.69% and 51.49% of the cells in CAP60 and CAP120, respectively, and the progression is consistent with the morphological results observed by TEM.

### 2.8 Apoptosis is initiated by excessive and instant oxidative stress

The CAP treatment increases the ROS content in RA‐FLS as demonstrated by flow cytometry (Figure 4c). The intensity of fluorescein isothiocyanate (FITC) shifts to the right for the CAP60 group and CAP120 group compared to the control (no treated) group. The median fluorescence intensity (MFI) representing the intracellular ROS content is compared and MFI of the CAP120 group (50146.33 ± 1243.48) is significantly higher than that of the control (no treated) group (32,502 ± 927.11) (*p* < 0.01) (Figure 4c). Since ROS in a low concentration impose antiapoptosis effects but in a high concentration induces apoptosis,^31^ it is proposed that CAP triggers the programed suicide of RA‐FLS by instantaneously exerting excessive oxidative stress.

### 2.9 Apoptosis is associated with the mitochondrial pathway

Mitochondria is believed to play a central role in the oxidative stress‐initiated apoptosis.^32^ In the next step, we test the MMP (ΔΨm) to explore the mechanism (Figure 4d). The percentages of C2 and C4 represent the cells under better conditions and a reduced membrane potential, respectively.^33^ The scatter plot shows that by prolonging the CAP treatment, the cell community tend to move from C2 to C4 and the ratio of cells with normal MMP decreases from 88% for the control (no treated) to 81% and 70% for CAP60 and CAP120, respectively (Figure 4d). The results reveal that the CAP treatment causes permeabilization of the mitochondrial membrane verifying the mitochondrial pathway associated apopotosis.

To understand the cascade of events in the mitochondria‐associated apoptosis, the proapoptotic and antiapoptotic proteins are semi‐quantified. As shown in Figure 4e, the western blot analysis shows that Bcl‐2 playing the antiapoptotic role is downregulated, while Bax as the proapoptotic member is upregulated. The Bcl‐2/Bax ratio drops by six times from 1.5 for the control (no treated) to ~0.25 for CAP120. As a result, Bcl‐2 cannot restrain Bax thus triggering apoptosis^34^ and accelerating mitochondrial outer membrane permeabilization as demonstrated by the test. The expression of the downstream proteins including cytochrome c and activated cleaved caspase‐3 (caspase‐3) exhibits an upregulated trend with prolonged treatment by CAP. This can be explained by that higher mitochondrial outer membrane permeabilization promotes release of cytochrome c and plays a key role in the formation of apoptosome.^35^, ^36^ The apoptosome then creates a platform to bring together molecules of the initiator caspase, among which caspase‐3 acts as an executioner cleaving other substrates within the cells.^37^ In short, apoptosis is activated by oxidative stress and initiated by the imbalanced expression of proapoptotic and antiapoptotic expression and involves the loss of MMP. Increase of ROS is an important reason for CAP‐induced apoptosis of the RA‐FLS cells.

## 3 DISCUSSION

There is more evidence showing that proliferative synovium plays an important role in the occurrence, development, and long‐term existence of RA joint lesions, and RA‐FLS constitutes the core effector cells in proliferative synovium.^38^, ^39^ Targeting RA‐FLS and inhibiting synovial hyperplasia are considered potential methods for treatment of RA, because they offer the advantages of potential improvement of the clinical efficacy and less impact on systematic immunity.^40^ The burgeoning strategy to ablate tumor based on plasma medicine inspires our attempt to treat RA with CAP. It has been validated that plasma technology has significant anticancer ability against cancer cell lines and subcutaneous tumors such as melanoma and liver tumors.^41^, ^42^, ^43^ When activated in the chronic inflammatory environment, RA‐FLS shares common characteristics with tumors that they can get rid of the growth restriction of contact inhibition, promote angiogenesis, and mitigate invasion of surrounding tissues,^44^ resulting in uncontrolled growth of synovium. Besides, as CAP is reported to selectively kill cancer cells by interfering with the mitotic cycle,^45^ RA‐FLS with increased mitotic activity also tends to be targeted by CAP without compromising normal tissues. It is verified by the in/ex vivo therapeutic effects shown in this work.

CAP has large potential in the treatment of RA based on the in/ex vivo studies. The in vivo trial shows that CAP can demolish both the typical symptoms and root causes of RA. Studies have shown that synovium exists in hypoxic environment for a long time. Oxidative stress caused by hypoxia changes the balance of intracellular redox and improves the survival ability of RA‐FLS under hypoxic conditions.^46^ CAP changes the stress intensity of surrounding tissues and intra‐articular environment to trigger the self‐antioxidant capacity, which is the fundamental reason for elimination of RA. As a result, symptoms including synovial hyperplasia, inflammatory infiltration and angiogenesis are never observed after treatment for 1 week. Ex vivo experiments also confirm this point and RA‐FLS as the dominant factor in RA is inactivated in terms of antiapoptosis, invasion, and migration capabilities.

**FIGURE 5:**
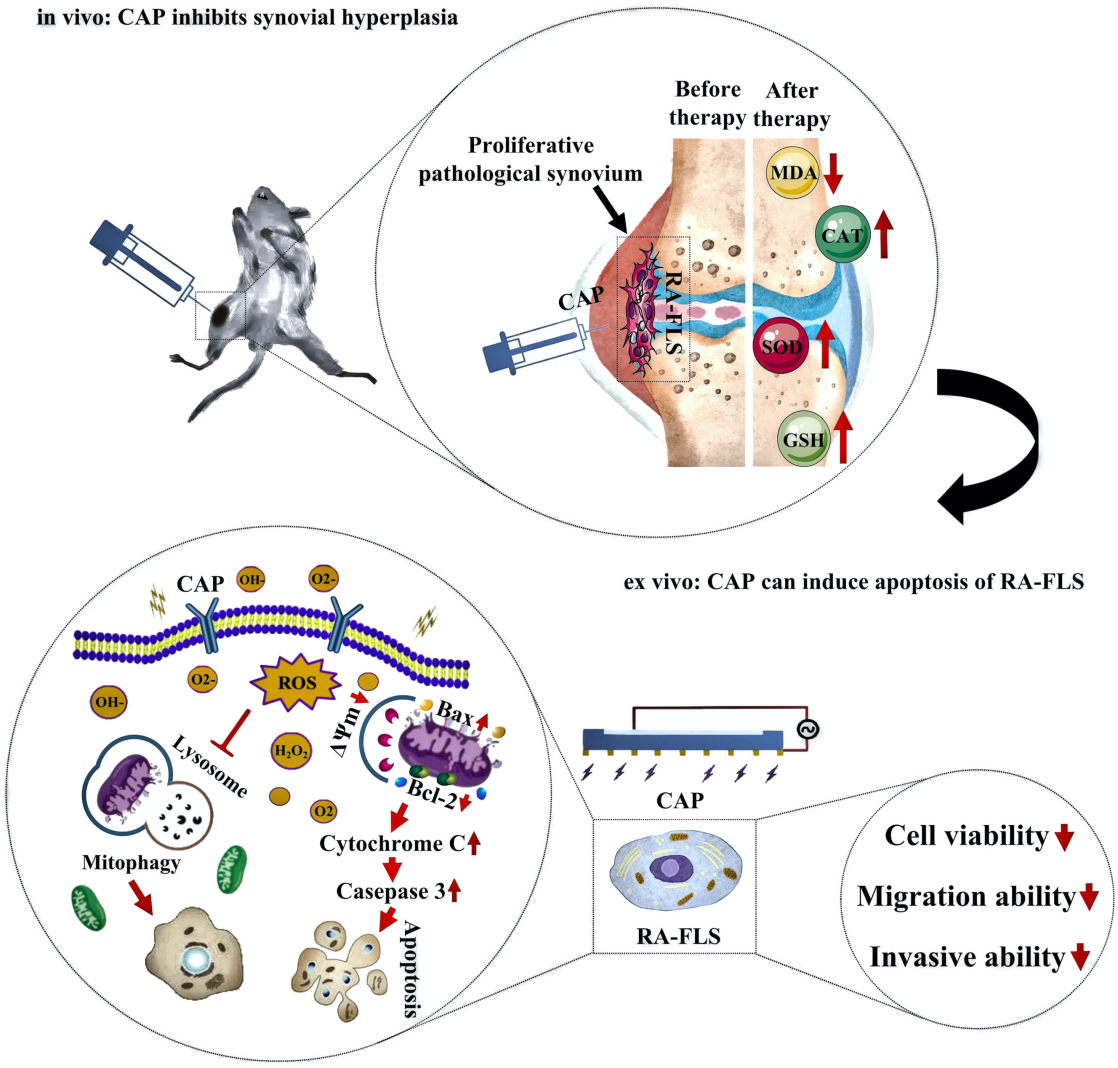
Schematic diagram of the mechanism of CAP's therapeutic effect on AIA rat joint synovium in vivo and ex vivo

Apart from the therapeutic effects, the underlying pathway is systematically analyzed by semi‐quantitatively monitoring the expression of critical proteins. The increased intracellular ROS in RA‐FLS triggers mitochondrial apoptosis by unbalancing the expression ratio of Bcl‐2/Bax, which devastates the membrane potential of mitochondria and cytochrome c is then released to the cytoplasm. The apoptosis program is fully incepted as verified by the upregulated expression of downstream caspase‐3, which acts as the executor responsible for proteolysis and pyrolysis of key proteins. This is consistent with previous works in which the plasma induces apoptosis of Candida utilis by triggering the mitochondrial pathway.^47^ In addition, Wang et al. have found that CAP can significantly inhibit migration and invasion of breast cancer cells consistent with our results.^48^ CAP interferes with the proliferation, remission, and invasion of RA‐FLS through the mitochondrial pathway and may be an effective way to inhibit the development of RA (Figure 5). ROS produced by CAP may act as a second messenger to activate the proapoptotic protein Bax in Bcl‐2 family through the signal transduction pathways such as P13k/AKT or MAPK/p38 and transfer it to the outer membrane of mitochondria to form oligomers.^49^, ^50^ It is related to the formation of the permeable transition pore (PTP). After PTP is formed, it will destroy the MMP to release cytochrome c into the cytoplasm.^51^ Cytochrome c can bind and activate caspase‐9 and further activate caspase‐3.^52^ Caspase‐3, which has been identified as the main executor of intracellular apoptotic response finally activates caspase‐3 to cleave effector proteins including PARP, induce DNA breakage in the nucleus, and finally lead to cell death.^53^ ROS and the caspase cascade apoptotic pathway not only cause apoptosis by mitochondrial dysfunction but also up‐regulate the Fas–FasL system through the exogenous apoptotic signal pathway, leading to activation of caspase‐8 and downstream caspase and producing apoptosis.^54^ CAP‐induced RA‐FLS cell apoptosis may also involve more complex mechanisms, but further studies are needed for confirmation.

In summary, inspired by the similarities between tumor and RA‐FLS, the therapeutic effects of CAP on the synovium of AIA rats are investigated by in/ex vivo experiments. CAP exerts spontaneous oxidative stress on the tissues. On the one hand, it triggers apoptosis of RA‐FLS and on the other hand, it activates the antioxidant ability of the surrounding tissues. Apoptosis of RA‐FLS contributes to the suspension of synovial hyperplasia and the antioxidant ability rescues the inflammatory and angioproliferative surrounding tissues to the normal state. The validated therapeutic effects on AIA rats indicate that CAP may become a suitable treatment for RA and stimulates the effects of CAP to treat tumor‐like diseases including cancer. The pathway also imparts important information necessary to understand the mechanism of plasma–tissue interactions.

## 4 MATERIALS AND METHODS

### 4.1 CAP‐i and CAP‐e equipment configuration

The CAP‐i device consists of a gas delivery device, a handle, and two electrode assemblies. The gas delivery device consists of a gas cylinder, a gas valve, and a gas flow meter that controls the amount and rate of gas input to the AIA rat joints. The two electrodes are assembled with an energized needle electrode and a grounded clip electrode is connected to a high‐voltage transformer. The needle electrodes are covered with an insulating film to prevent damage from direct contact with the tissues. The working voltage effective value of CAP‐i device is about 700 V and the current is about 3 mA.

The CAP‐e device is designed based on the area of a 96‐well cell culture plate with a length of 10 mm and a width of 15 mm. The CAP‐e device consists of the Al electrode, Al_2_O_3_ dielectric layer, and Cu electrode, where the Al_2_O_3_ dielectric layer is prepared by micro‐arc oxidation and the Cu electrode is prepared by plasma spraying. The detailed preparation process is described in our previous paper.^20^ The advantage of this design is to reduce leakage and energy consumption. The working voltage of CAP‐e is about 2 kV and the current is about 0.1 mA.

### 4.2 Intervention of CAP‐i on synovium of adjuvant‐induced arthritis rats

As early as 2005, Alexandre et al. reported that the treatment of intervertebral disc herniation by injecting oxygen‐ozone mixed gas into the intervertebral disc had a significant therapeutic effect on pain and nerve root dysfunction.^55^ Studies showed that intraarticular ozone therapy could effectively alleviate the pain of patients with osteoarthritis.^56^ Based on these studies, intraarticular gas injection was feasible and safe to some extent. The uppermost end of the puncture needle was connected to the electrode and the puncture needle was covered by insulating materials with the tip exposed only to the discharge as the gas was injected into the body. The other wire was grounded and fixed to the rats' legs. During the experiment, the rat was anesthetized and fixed to the plate with a rubber band. After the puncture needle was inserted into the rats' joints, the power and timer were on and by using the gas cylinder and flow valve, 2 ml of air was injected slowly into the joint cavity of the AIA rat. Finally, the plasma was generated around the joint synovium by arc discharge.

### 4.3 Establishment of the AIA rats' model and experimental design

Male SD rats' weighing 240 ± 20 g (7–8 weeks old) were obtained from the Experimental Animal Center of Anhui Medical University and all animal care and treatment procedures were approved by the Animal Ethics Committee of Anhui Medical University. The experimental rats were divided randomly into four groups (eight rats in each group): normal group, AIA group (untreated), CAP 60 (The CAP treatment durations were 60 s), and CAP 120 (The CAP treatment durations were 120 s). After adaptive feeding at room temperature for 1 week without abnormality, all the rats were anesthetized with chloral hydrate (3:1 mg/kg, intraperitoneal). The AIA rats' model was established by subcutaneous injection of the complete Freund' s adjuvant (Chondrex, USA) at the left rear toe (injection dose: 0.1 ml/100 g). The rats in the normal group were injected with the same amount of normal saline. After modeling, the rats were fed with a normal diet and underwent normal activities for 21 days. On the 21st day, the treatment group was treated with CAP at the knee joint for 60 s or 120 s, once every other day within 7 days for a total of four times. The rats in normal group and AIA group were not treated. The CAP treatment was completed on the 27th day and 24 h after the last CAP treatment, all rats were sacrificed. The whole experiment lasted for 28 days.

### 4.4 Hematoxylin–Eosin staining

AIA rats were killed with overdose of chloral hydrate after CAP treatment, and the synovial tissue of the left knee joint was taken out. The specific procedures for HE staining of the synovial tissue of the knee joint are as follows:

1. Fixation: Fresh synovial tissue was put into 4% paraformaldehyde and fixed at 4°C for 48 h, and the fixed sample was dehydrated in ethanol solution of different concentrations.
2. Paraffin embedding and slicing: put the transparent synovial tissue block into the melted paraffin for complete embedding and cut the cooled paraffin block into 4 μM thick slice.
3. Dewaxing and dyeing: the slices are dewaxed with xylene, washed with high concentration to low concentration alcohol and finally washed with distilled water. Hematoxylin aqueous solution and alcohol eosin staining solution are used successively.
4. Dehydration and sealing: the stained sections are dehydrated by alcohol, and then made transparent by xylene. Finally, seal with neutral glue.

### 4.5 Doppler ultrasound

On the 28th day, the knee joints of rats in the normal group, AIA group, CAP60 group, and CAP120 group were examined by the Doppler ultrasound instrument (Canon model, Japan, aplio i800 tus‐ai800). The experimental rats were fixed on the operating table in the supine position. The hair on both the knees of the rats was removed and the knee joint remained in the flexion position. The skin on the surface of the knee joint was evenly coated with the coupling agent and the ultrasonic probe was placed in the joint space of the knee joint to observe the blood flow in the synovium.

### 4.6 Oxidative stress level

After the rats were sacrificed on the 28th day, the synovial tissue was obtained from the left knee joint and added with nine times the volume of normal saline with the proportion of weight (g): Volume (ML) = 1:9 to prepare the 10% tissue homogenate at 2500 rpm for 10 min using a commercial kit (Nanjing Jiancheng Bioengineering Institute, China). The levels of malondialdehyde (MDA, A003‐1), superoxide dismutase (SOD, A001‐3), catalase (CAT, A007‐1‐1), and reduced glutathione (GSH, A006‐2‐1) in the tissue homogenate were determined according to the manufacturer's protocol.

### 4.7 Cell culture and treatment

RA‐FLS was purchased from Shanghai Biyuntian Biotechnology Co., Ltd. The cells were cultured in DMEM (Servicebio, China, G4510) medium containing 20% fetal bovine serum (TIANHANG, China, 11011‐8611), 100 U/ml penicillin, and 100 U/ml streptomycin in a 5% CO_2_ humidified incubator (Lishen, China, HF‐90) at 37 °C. The suspension containing RA‐FLS (cell concentration 1 × 10^6^/ml) was added to a 96‐well culture plate at 100 μl per well and culturing continued for 12 h. The culture medium was removed before the intervention. The 96‐well culture plate was placed under the CAP‐e equipment with a distance of 5 mm (the working voltage effective value of CAP‐e is about 2 kV and the current effective value is about 0.1 mA), and then treated for 0, 60, and 120 s (recorded as control [no treated], CAP60, CAP120 respectively). After treatment, the cells were cultured for 24 h and collected for subsequent experiments.

### 4.8 Cell Counting Kit‐8 analysis and inverted microscopy

The cells were inoculated in 96‐well culture plates with 3 × 10^3^ cells per well and incubated overnight, and then the cells were treated with CAP60 s and CAP120 s. After the treatment, the cells were cultured for 24 h. After reaching the time point, 10 μl of CCK‐8 (Wanleibio, China, WLA074) was added to each well and incubated at 37°C in a 5% CO_2_ incubator for 2 h. The OD value at 450 nm was measured on a microplate reader and the cell morphology was observed under an inverted microscope (OLYMPUS, Japan, IX53).

### 4.9 Wound healing assay

The cells of each group were cultured and the medium was changed to a serum‐free one before the experiment followed by addition of 1 μg/ml mitomycin C (Sigma, USA) for 1 h. A 200 μl pipette tip was used to create cell scratching. The cell surface was rinsed with the serum‐free medium once to remove debris and the cells were cultured in the incubator at 37°C under 5% CO_2_ for 0 and 24 h respectively, before microscopic (OLYMPUS, IX53) examination to calculate the migration distance.

### 4.10 Transwell experiments

The Transwell chamber (Corning, USA, 354234) was employed to analyze cell invasion. The cells of each group were cultured to a cell density of about 90% and put into the serum‐free medium. The cells were counted and diluted to prepare the cell suspensions. The coated Transwell chamber was put on a 24‐well plate with 800 μl of the culture medium containing 10% FBS added to the lower chamber and 200 μl of the cell suspension to the upper chamber. The number of cells was 3 × 10^4^/well. The 24‐well plate was cultured in an incubator at 37°C under 5% CO_2_ at the saturated humidity for 24 h. The Transwell cell was washed twice with PBS, fixed at room temperature with 4% paraformaldehyde for 25 min, stained with 0.4% crystal violet for 5 min, and rinsed with distilled water. The cells invading the lower layer of microporous membrane were counted by an inverted microscope. Five fields were chosen from each sample to count the cells and averages were calculated.

### 4.11 Transmission electron microscopy

A total of 1 × 10^6^ cells were fixed in 2.5% pentanediol precooled at 4°C for 12 h. After rinsing with PBS, the cells were fixed in 1% osmic acid for 1.5 h, dehydrated in 30% and 50% ethanol for 10 min, stained with 70% ethanol uranium acetate for 3 h, dehydrated in 80% ethanol for 10 min, 95% ethanol for 15 min, and 100% ethanol twice for 50 min, and finally dehydrated in propylene oxide for 30 min. After embedding in epoxy resin, it was put in a 45°C oven for 12 h and 72°C oven for 24 h. The embedded block was cut into 70 nm thick sections (Leica, Germany). The cell image after staining was obtained by transmission electron microscopy (Electronics, Japan) and also a digital camera (Munster, Germany).

### 4.12 Apoptosis assay

Apoptosis was analyzed by flow cytometry using the annexin V‐FITC apoptosis detection kit (Wanleibio, China, WLA001). The cells of each group were collected, centrifuged at 300 rpm for 5 min and washed twice with PBS. The supernatant was discarded and a binding buffer with a volume of 500 μl was then added. The 5 μl of annexin V‐FITC and 10 μl of propidium iodide were added and mixed. The cells were incubated without light for 15 min before flow cytometer (ACEA BIO, USA, NovoCyte) was performed.

### 4.13 Intracellular reactive oxygen species contents

The content of ROS in RA‐FLS cells was measured by the average fluorescence intensity of DCFH‐DA before and after CAP intervention. The cells of each group were collected and the cell concentration was adjusted to 1 × 10^6^ cells/ml. DCFH‐DA (10 μmol/L) was added and incubated at 37°C for 20 min and mixed every 3 min so that the cells would fully function and the cells without staining served as the negative control. The cells were washed with a serum‐free medium to remove DCFH‐DA that did not enter the cells. Finally, the cells in each group were tested by flow cytometry and the data were analyzed by FlowJo VX software to obtain the MFI value.

### 4.14 JC‐1

The cells were collected, washed twice with PBS, and resuspended with 500 μl of the JC‐1 staining solution. The cell density was about 10 × 10^5^/ml. They were incubated for 20 min under 5% CO_2_ at 37°C and centrifuged at 600 rpm for 4 min. The cells were washed with the JC‐1 staining buffer twice and resuspended with a proper amount of JC‐1 staining buffer for subsequent flow cytometry.

### 4.15 Western blotting

The cells were lysed with the Ripa buffer (Beyotime, China, P0013B) and centrifuged (4°C, 12,000 rpm, 10 min) before the supernatant was absorbed to obtain the cell protein. A 25 mg/ml of protein standard was prepared by adjusting the concentration to be the consistent with Ripa, mixed evenly with the protein‐loading buffer and put in a water bath at 100°C for 10 min to denature the protein. The protein sample was loaded into the SDS‐PAGE gel sample hole for electrophoresis and the PVDF membrane with the same size was used by electrophoresis. The PVDF membrane was immersed in skim milk and sealed at room temperature for 2 h before immersion in Bcl‐2 (abcam, Britain, ab32124), Bax (abcam, Britain, ab32503), caspase 3(abcam, Britain, ab184787), and cytochrome c (Bioworld, USA, Bs1089) primary antibody, incubated overnight at 4°C, and washed three times with PBST for 15 min each. The PVDF membrane was then immersed in the secondary antibody working solution (secondary antibody: sealing solution = 1:5000) for 1 h and washed three times with PBST for 15 min. Finally, the protein content was derived using the Saimer Fischer ECL kit and ImageJ software.

### 4.16 Statistical analysis

The data generated in the experiments were presented as mean ± standard deviation (SD) and all the data were analyzed statistically by the IBM SPSS software version 23.0 (SPSS). Multigroup data were compared by one‐way ANOVA and multiple comparison among groups (ANOVA) was performed by the LSD test. The independent sample *t*‐test was employed to compare the data between the two groups. The *p* value refers to the statistical probability with *p* < 0.05 indicating statistically significant difference.

## AUTHOR CONTRIBUTIONS

**Chengbiao Ding:** Funding acquisition (equal); investigation (equal); software (lead); writing – original draft (lead). **Leying Ni:** Investigation (equal); methodology (equal); software (equal); validation (equal); visualization (equal); writing – original draft (equal). **Qi Liu:** Data curation (equal); investigation (equal); methodology (equal); visualization (equal); writing – original draft (equal). **Chenxu Zhou:** Investigation (supporting); software (supporting); visualization (supporting). **Guomin Wang:** Project administration (equal); supervision (equal); writing – review and editing (equal). **Paul K. Chu:** Conceptualization (equal); funding acquisition (equal); writing – review and editing (equal). **Zhengwei Wu:** Conceptualization (lead); funding acquisition (lead); project administration (lead); resources (lead); supervision (lead); writing – review and editing (lead).

## FUNDING INFORMATION

This work was supported by the National Natural Science Foundation Incubation Program of the Second Hospital of Anhui Medical University (2020GMFY06), the 2022 Natural Science Foundation of Anhui Province (C.B.D), the Key R & D plan of Anhui Province (201904a07020013), the Natural Science Foundation for Young People of Anhui Medical University (2019XKJ036), Collaborative Innovation Program of Hefei Science Center (CAS, CX2140000018), the Quality Engineering Project of Anhui Province (2019JYXM0999), the Shenzhen ‐ Hong Kong Innovative Collaborative Research and Development Program (SGLH20181109110802117 and CityU 9240014), the City University of Hong Kong Donation Research Grant (DON‐RMG No. 9229021), the Health and Medical Research Fund, the Food and Health Bureau, the Government of the Hong Kong Special Administrative Region (No. 9211320), the Funding for Joint Lab of Applied Plasma Technology (JL06120001H) and HK Tech 300 Seed Fund (SF202109169).

## CONFLICT OF INTERESTS

The authors declare no competing interests.

## Data Availability

All data needed in the paper are present in the paper. Additional data related to this paper may be requested from the authors.
